# Assessment of the in vitro growing dynamics and kinetics of the non-pathogenic J and pathogenic 11 and 232 *Mycoplasma hyopneumoniae* strains

**DOI:** 10.1186/s13567-018-0541-y

**Published:** 2018-05-25

**Authors:** Beatriz Garcia-Morante, Arkadius Dors, Rocio León-Kempis, Ana Pérez de Rozas, Joaquim Segalés, Marina Sibila

**Affiliations:** 1 IRTA, Centre de Recerca en Sanitat Animal (CReSA, IRTA-UAB), Campus de la, Universitat Autònoma de Barcelona, 08193 Bellaterra, Spain; 2 Boehringer Ingelheim Veterinary Research Center GmbH & Co. KG (BI VRC), 30559 Hannover, Germany; 3 Department of Swine Diseases, National Veterinary Research Institute, 24-100 Puławy, Poland; 4 UAB, Centre de Recerca en Sanitat Animal (CReSA, UAB-IRTA), Campus de la, Universitat Autònoma de Barcelona, 08193 Bellaterra, Spain; 5 Departament de Sanitati Anatomia Animals, Facultat de Veterinària, UAB, 08193 Bellaterra, Spain

## Abstract

Information on the in vitro growth of pathogenic and non-pathogenic *Mycoplasma hyopneumoniae* (*M. hyopneumoniae*) strains is scarce and controversial. Despite its limitations, the colour changing units (CCU) assay is still considered the golden standard titration technique for *M. hyopneumoniae* culture. Thus, the aims of the present study were: (1) to describe the growth dynamics and kinetics of pathogenic and non-pathogenic *M. hyopneumoniae* strains, and (2) to monitor the strains’ daily growth by ATP luminometry, CCU, colony forming units (CFU), and DNA quantification by real time quantitative PCR (qPCR) and by fluorescent double-stranded DNA (dsDNA) staining, to evaluate them as putative titration methodologies. The growth of the non-pathogenic J (ATCC^®^25934™) type strain and the pathogenic 11 (ATCC^®^25095™) reference strain and 232 strain was modelled by the Gompertz model. Globally, all three-strain cultures showed the same growing phases as well as similar maximal titres within a particular technique, but for CFU. However, the J strain displayed the fastest growing. During the logarithmic phase of growing, CCU, ATP and *M. hyopneumoniae* copy titres were strongly and linearly associated, and correlation between techniques could be reliably established. In conclusion, real-time culture titration by means of ATP or molecular assays was useful to describe the in vitro growth of the tested strains. Knowledge about the in vitro growth behaviour of a specific strain in a specific medium may provide several advantages, including information about the time required to reach maximal titres by the culture. Noteworthy, the obtained results refers to the three strains used, so extrapolation to other *M. hyopneumoniae* strains or culture conditions should be made cautiously.

## Introduction

In 1965, Maré and Switzer in the United States and Goodwin in the United Kingdom isolated *Mycoplasma hyopneumoniae* (*M. hyopneumoniae*) strains 11 and J, respectively [1]. Since then, this bacterium has been reported to play a prominent role in pig respiratory diseases [2]. Strain J was accepted as the type strain of *M. hyopneumoniae* [1]. Although originated from lung lesions and considered pathogenic, pneumonia was not reproduced when pigs were inoculated with this strain after several in vitro passages [3, 4]. From then, J strain was recognized as non-pathogenic. Additionally, some of the current commercial bacterins used to control enzootic pneumonia are based on J strain [5]. On the other hand, *M. hyopneumoniae* strain 232, which was isolated from serially passaged porcine lung homogenate containing strain 11 [3], is widely used in experimental studies in the United States and has progressively substituted strain 11 in multiple studies, though both are considered pathogenic strains [3]. *M. hyopneumoniae* strain 11 is also used as inactivated whole cell concentrate for vaccination [6].

Despite comparative genomic [7–9] and proteomic [10, 11] analyses between *M. hyopneumoniae* strains have been made, little is still known on the in vitro growth of strains differing in pathogenicity and, by extension, in virulence. In addition, available information in this respect may result contradictory. On the one hand, a higher virulence of *M. hyopneumoniae* has been associated with a faster in vitro growth [12], though this could not be corroborated afterwards [13]. On the other hand, low-passaged *M. hyopneumoniae* strains may grow slowly and may yield lower number of cells in medium than higher-passaged strains, which, in turn, may not be virulent [3, 14, 15]. In connection with this matter, non-pathogenic strains’ protein expression profile has been shown to be more related to metabolism than to infectiousness [10, 11]. Therefore, the putative relationship between pathogenicity and in vitro growing capacity of *M. hyopneumoniae* deserves further investigation.

Besides, outputs obtained from live animals inoculated with *M. hyopneumoniae* may vary considerably from one experiment to another [16–19]. Although many other factors might be involved, an inoculum dose-dependent response has been reported in these experiments [20]. Despite the well-known colour changing units (CCU) assay limitations in the assessment of the live *M. hyopneumoniae* cell titres [13, 21], this technique continues to be the most frequently used to calculate the inoculum dose given to the animals under experimental settings. Indeed, ATP luminometry [13] and flow cytometry [21] assays have been proposed as more reliable, accurate and as real-time titration tools [13, 21]. Notwithstanding, none of these two techniques is specific for *M. hyopneumoniae*. Therefore, a deeper comparison of the CCU with other real-time, accessible and, if possible, *M. hyopneumoniae*-specific techniques remains of general interest.

The aims of the present work were twofold. Firstly, growth dynamics and kinetics of pathogenic (11 and 232) and non-pathogenic (J) strains *M. hyopneumoniae* were described. Secondly, bacterium growth was monitored by different methodologies, including ATP luminometry, CCU and colony forming units (CFU), and molecular detection methods as real time quantitative PCR (qPCR) and fluorescent double-stranded DNA (dsDNA) staining, with the objective to evaluate them as putative techniques for titration of *M. hyopneumoniae* culture.

## Materials and methods

### *M. hyopneumoniae* strains and medium for culturing

Type strain J (ATCC^®^25934™) and strain 11(ATCC^®^25095™) were obtained from the American Type Culture Collection (ATCC). Boehringer Ingelheim Veterinary Research Center (BIVRC) GmbH & Co. KG (Hannover, Germany) kindly provided strain 232. All strains were grown in the ATCC recommended conditions: ATCC medium for *M. hyopneumoniae* culture purposes (ATCC^®^ Medium 1699: Revised Mycoplasma medium) at 37 °C in an aerobic or 5% CO_2_ atmospheres for broth and solid mediums, respectively.

### Experimental design

In order to compare the growth curves of these three *M. hyopneumoniae* strains, an initial inoculum of each of them was prepared from a single colony. Thus, a well-shaped and well-separated colony of each strain was selected with the help of an optical microscope to localize it on the plate. Thereafter, plates were opened under the laboratory hood and agar was punctured with a sterile 1000 μL size filter pipette tip and immediately resuspended in 1 mL of ATCC broth medium. After approximately 2 weeks of incubation, these cultures turned from red to orange to yellow and were then frozen at −80 °C until used. These frozen cultures were considered the initial inoculums.

For the experiment, *M. hyopneumoniae* strain (J, 11 and 232) initial inoculums were thawed and 100-fold diluted in duplicates (named A and B) in a final volume of 45 mL of ATCC broth medium. A tube containing only 45 mL of broth served as a control. Cultures were grown statically and followed up until the senescence phase was reached. Thus, once the initial inoculum dilutions were done, 2 vials of 1 mL each of every culture replicate and control were taken at day 0 post-culture inoculation (D0), at 12 h after (D0.5) and, afterwards, every 24 h until the end of the study (D14). In order to avoid cross-contamination between *M. hyopneumoniae* strains, manipulation of each strain culture was performed in separate laboratory hoods. One vial of each duplicate was directly frozen at −20 °C whereas the other one was freshly and daily used for ATP luminometry, CFU and CCU titre assessments. The frozen vial was used once the experiment was finished for genomic DNA extraction and *M. hyopneumoniae* copies calculation by real time quantitative PCR (qPCR) and fluorescent double-stranded nucleic acid stain.

### CCU assay

Formerly described conditions were followed to assess the number of CCU [13] at every single time point. Briefly, tenfold serial dilutions (until 10^−11^) of each *M. hyopneumoniae* strain culture replicate in ATCC broth medium were done. Microtiter plates were then sealed with plastic foil, and incubated at 37 °C for 2 weeks. The CCU titre was determined from 2 repeated measurements (A and B) per each strain culture (but for control) and expressed as mean CCU/mL.

### CFU assay

At every time point, solid medium plates were inoculated with each strain culture replicate. Plates were divided into eight equal parts. In each segment, a drop of 10 μL of either fresh undiluted culture or a tenfold serial dilution in ATCC broth medium (from 10^−1^ to 10^−7^) was spotted onto. In the case of the control, no dilutions were performed and the broth was directly spotted onto the plate. Drops were allowed to become air-dried under the laboratory hood and plates were covered and incubated for 7 days. By means of an optical microscope, *M. hyopneumoniae* colonies were counted until the last dilution in which they were present. The counts of each two strain replicates (A and B) were considered and the mean CFU/mL calculated.

### ATP dependent luminometry assay

For luminometry, the BacTiter-Glo™ Microbial Cell Viability assay (Promega, Madison, WI, USA) together with a Fluoroskan Ascent^®^ FL luminometer (Thermo Electron Inc., Milford, MA, USA) were used. For every time point measurement, 100 μL of freshly *M. hyopneumoniae* cultures and control were taken and mixed with 100 μL BacTiter-Glo™ reagent in white opaque 96-well microplates (PerkinElmer, Waltham, MA, USA). Directly after, the contents were mixed briefly by orbital shaking and incubated at room temperature for 5 min. Luminescence was thereafter measured and recorded as relative light units (RLU). To transform RLUs into ATP concentrations, tenfold dilutions of r-ATP (Promega, Madison, WI, USA) in ATCC broth medium, from 10 µM to 1 nM, were used as standard. For each plate, sample results were adjusted by subtracting the mean RLUs of the blank (ATCC broth medium). ATP titres of each strain culture were determined as the mean of the replicate measurements (A and B) and expressed as pmol ATP/mL of culture of *M. hyopneumoniae*.

### Genomic DNA extraction

DNA was extracted for every time point from 1 mL of broth culture of each strain replicate and control using GenElute™ Mammalian Genomic DNA Miniprep Kit (Sigma-Aldrich, Saint Louis, MO, USA). Once thawed, cultures and control were centrifuged at 10 000 *g* during 2 min and the cell pellet was then resuspended with 200 μL of the provided resuspension solution. DNA was eluted in 200 μL of elution buffer.

### Fluorescent double-stranded DNA stain assay

DsDNA content was determined using the Quan-iT™ PicoGreen^®^ dsDNA kit (Life Technologies, Eugene, OR, USA) together with a Fluoroskan Ascent^®^ FL fluorimeter (Thermo Electron Inc., Milford, MA, USA). Ten microliter of the extracted DNA

from every time point and strain replicates and control was mixed with 190 μL of Quan-iT™ PicoGreen reagent in white opaque 96-well microplates (PerkinElmer, Waltham, MA, USA). Afterwards, the contents were mixed briefly by orbital shaking and incubated at room temperature for 5 min. Fluorescence was then measured and recorded as relative fluorescence units (RFU). In order to convert RFUs into dsDNA concentrations, tenfold dilutions of the provided Lambda DNA standard in the elution buffer used for the DNA extraction (GenElute™ Mammalian Genomic DNA Miniprep Kit) were used to elaborate a standard curve (final concentrations from 500 to 0.5 ng/mL). Samples’ fluorescence value was adjusted by subtracting the mean RFU of the reagent blank (elution buffer). DsDNA concentration of each strain culture was obtained as the mean of the replicate measurements (A and B) and expressed as ng/mL. Such concentration was used thereafter for determining the *M. hyopneumoniae* copy numbers per mL in each template.

### Real time quantitative PCR for *M. hyopneumoniae*

A previously described qPCR specific for *M. hyopneumoniae* was performed [22]. DNA extracted from cultures was used as template and plasmid as a standard for the qPCR development. Since sensitivity of the aforesaid qPCR was reported to be between 10^3^ and 10^4^ *M. hyopneumoniae* copies/mL of plasmid [22], all those samples with a bacterial load below 10^4^ *M. hyopneumoniae* copies/mL were considered negative. QPCR results were expressed as the mean of *M. hyopneumoniae* copy numbers per mL of culture of the replicate measurements (A and B).

### *M. hyopneumoniae* strains growth modelling

In order to quantitatively describe the in vitro growth of *M. hyopneumoniae* strains, a non-linear asymptotic model, namely the Gompertz equation, was fitted to each *M. hyopneumoniae* strain culture titre data obtained from the daily application of the different evaluated methodologies. The Gompertz equation enables to calculate growth kinetic parameters, thereby allowing the comparison of these parameters between the different techniques [23]. The aforesaid model was fitted to data with STATISTICA version 8.0 (Stat Soft, Inc., USA) and the equation used can be written in the form [24]: *Y* = A*(e(− B*e(− C*t))).

The dependent variable *Y* represents the observed titre value at a time (*t*); parameter *A* describes the titre value as it approaches infinity, though representing the maximal titre; parameter *B* is an integration constant defined as the proportion of the asymptotic maximal titre obtained after the initial inoculum dilution (established by the initial titre value and *t*), and parameter *C* equals the ratio between the maximum growth rate (*μ*_max_) and the maximal titres and determines the slope of the curve. Thus, *μ*_max_ was obtained by multiplying *A* and *C* parameters. Considering *M. hyopneumoniae* as an asynchronic culture (i.e. cells of the total population do not divide simultaneously), mean generation time (*G*) was estimated by l/*μ*_max_ as proposed by Baranyi and Roberts [25].

Model goodness of fit was evaluated according to the *R*^2^ and the average prediction error (APE). The first determines the percentage of variation in *Y* explained by the statistical model whereas the latter quantifies the relative disagreement between observed (*Y*) and predicted (*PY*) titre values for each specific *t* and is calculated as follows: APE % = ([*Y* − *PY*]/*Y*) × 100 [26]. In order to evaluate the methodology from which the Gompertz growth model was better fitted to the data, mean APE per technique was calculated by averaging values obtained from each strain culture.

Statistical analyses were performed using STATISTICA version 8.0 (Stat Soft, Inc., USA). Differences between each *M. hyopneumoniae* strain mean value of the parameters obtained from the Gompertz modelling were tested for statistical significance by Kruskal–Wallis test (non-parametric, one-way ANOVA) with post hoc multiple comparisons (Dunn’s multiple comparison test). Normal distribution and the constant variance were evaluated by the Shapiro–Wilk’s test and Levene’s test, respectively. *p* values < 0.05 were considered to be significant.

### Relationship between assessed techniques

In order to compare the different assessed methodologies, a linear regression analysis between each pair of *M. hyopneumoniae* culture titration techniques was carried out on the log2 transformed data during each strain maximal culture growth, namely logarithmic (log) phase. Thus, the period of study days compared was established taking into account the log phase duration from the growth response determined by the ATP assay in each strain culture. From such analysis, regression formulae were acquired to enable conversion of data (culture titre) obtained by means of different techniques. All statistical analyses were carried out using STATISTICA version 8.0 (Stat Soft, Inc., USA). The significance level (*p*) was set at 0.05 with statistical tendencies reported when *p* < 0.10.

## Results

Negative control (uninoculated ATCC broth medium) had similar ATP values and dsDNA fluorescence reactions than the blank throughout the study (from D0 to D14). Additionally, no colonies were found in any of the agar plates inoculated, no colour change was detected at any time point and qPCR results were also negative. Unfortunately, a fungus-like contamination was detected in replicate A of *M. hyopneumoniae* J strain culture at D7. Therefore, from D7 onwards, different methodology titres were assessed for J strain culture only from the replicate B. Microbial growth curves obtained for each strain and each technique were log2 transformed for the purposes of graphic representation.

### *M. hyopneumon*i*ae* ATP growth response curves

**Figure 1:**
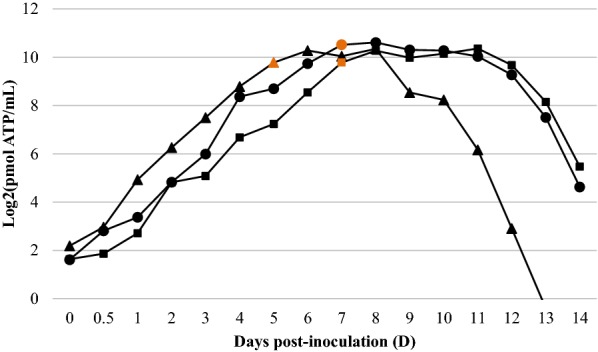
**ATP growth curves of** ***M. hyopneumoniae*** **strains J (filled triangle), 11 (filled square) and 232 (filled circle).** Data points represent means of the 2 replicate ATP luminometry measurements (A and B) within each strain. The orange data points indicate when the original medium colour change was first visually detected in each strain culture.

The growth response determined by ATP for each of the three *M. hyopneumoniae* strains is represented in Figure 1. It is important to mention that ATP concentration levels between strain initial inocula after thawing were very similar (1222.1 ± 86.2 pmol ATP/mL), which verified that the growth dynamics was started from approximated equal conditions for each *M. hyopneumoniae* strain culture. By means of ATP luminometry, all strains showed growth curves with log, stationary and death phases, and culture colour change (from the original red to orange) firstly detected at the end of the logarithmic phase; when yellow, cultures were established in the stationary phase. *M. hyopneumoniae* J strain reached earlier the stationary phase than 11 and 232 strains. Nonetheless, the latter ones had longer stationary phases followed by less sharp senescence phases in comparison with J strain. Additionally, all three evaluated strains reached similar maximal raw ATP concentrations (1395.5 ± 148.9 pmol ATP/mL) once in the stationary phase.

### *M. hyopneumon*i*ae* CCU growth response curves

**Figure 2:**
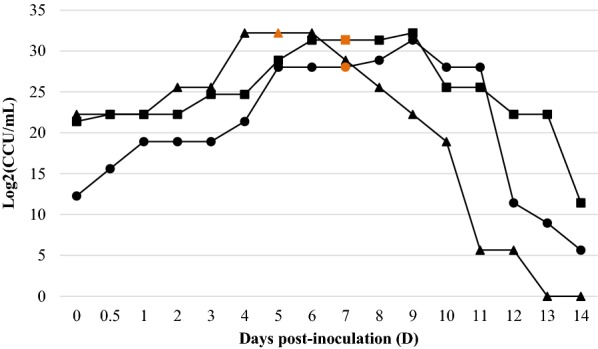
**CCU growth curves of** ***M. hyopneumoniae*** **strains J (filled triangle), 11 (filled square) and 232 (filled circle).** Data points represent means of the 2 replicate CCU assay measurements (A and B) within each strain. The orange data points indicate when the original medium colour change was first visually detected in each strain culture.

The growth response determined by the CCU assay for each of the three *M*. *hyopneumoniae* strains is represented in Figure 2. In contrast to ATP luminometry, a lag phase was observed for J and 11 strains. Following such an initial phase, log, stationary and death phases could also be visually differentiated, although not as easily as with ATP concentration along time. Importantly, when all strain cultures colour shift was detected, CCU maximal titres were already reached. Similarly to ATP, all three evaluated strains reached similar maximal raw CCU titres (4.25 × 10^9^ ± 1.30 × 10^9^ CCU/mL) in the stationary phase. Those were also reached earlier by J strain, which again showed a shorter stationary phase than *M. hyopneumoniae* 11 and 232 strains.

### Growth of *M. hyopneumoniae* on solid media

**Figure 3:**
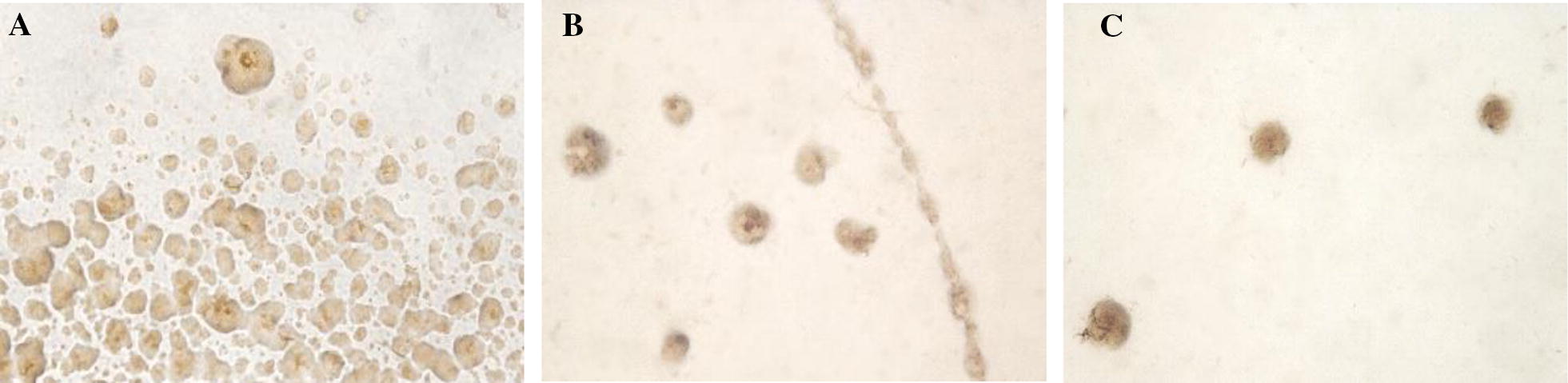
**Colonies of** ***M. hyopneumoniae*** **J strain on solid media.** Colonies were photographed at 5 days post-inoculation (D5) of **A** undiluted culture, **B** 10^−1^ and **C** 10^−2^ dilution of the culture in ATCC medium. In **B**, the limit of the culture drop can be differentiated with the colonies observed within such limit. Once inoculated, solid plates were incubated for 7 days at 37 °C and 5% CO_2_ and photographed using a microscope at ×40.

**Figure 4:**
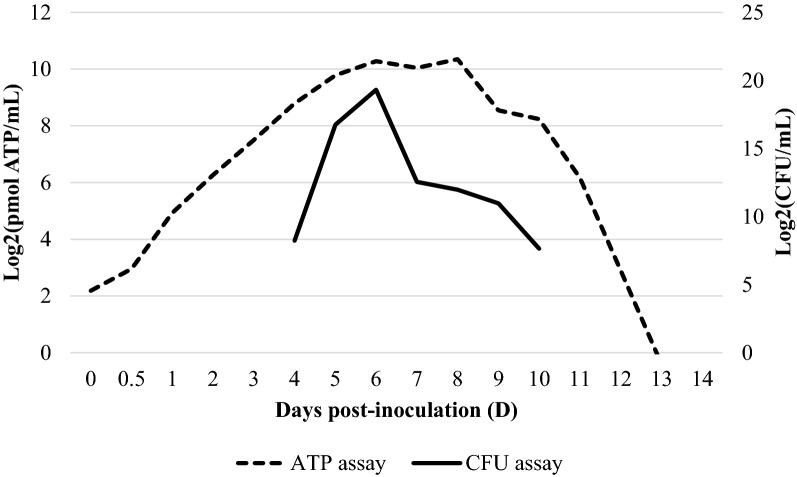
**Counts of CFU and ATP concentration per millilitre of** ***M. hyopneumoniae*** **J strain culture.** The continuous line represents the counts of CFU per millilitre whereas the discontinuous line depicts the ATP concentration per millilitre along the study period.

In this experiment, solid medium enabled the growth of *M. hyopneumoniae* J, 11 and 232 strains. Colonies from such three strains were phenotypically indistinguishable between them through microscopic visualization. Thus, end-point colonies ranged in size, from 100 to 240 µm, and were rough with irregular margin, normally lacking a clearly defined nipple (no “fried egg appearance”) (Figure 3). Colonies were observed as early as D3 for J strain and at D6 and D8 for 11 and 232 strains, respectively. The period of time in which colonies were observed varied between 7 days for J, 3 for 11 and 2 for 232 strains. Hence, the growth curve using viable counts on the solid medium could only be determined by J strain. Since all the growth phases could be well established by means of ATP luminometry, counts of CFU per mL of *M. hyopneumoniae* J strain to its ATP growth response along the study period is depicted in Figure 4.

### Copies of *M. hyopneumoniae* genomic DNA determination

**Figure 5:**
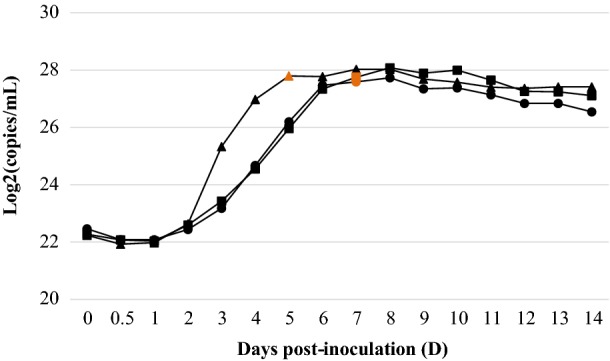
***Mycoplasma hyopneumoniae*** **strains J (filled triangle), 11 (filled square) and 232 (filled circle) copies per millilitre of dsDNA.** Data points represent means of the 2 replicate fluorimetry measurements (A and B) within each strain. The orange data points indicate when the original medium colour change was first visually detected in each strain culture.

**Figure 6:**
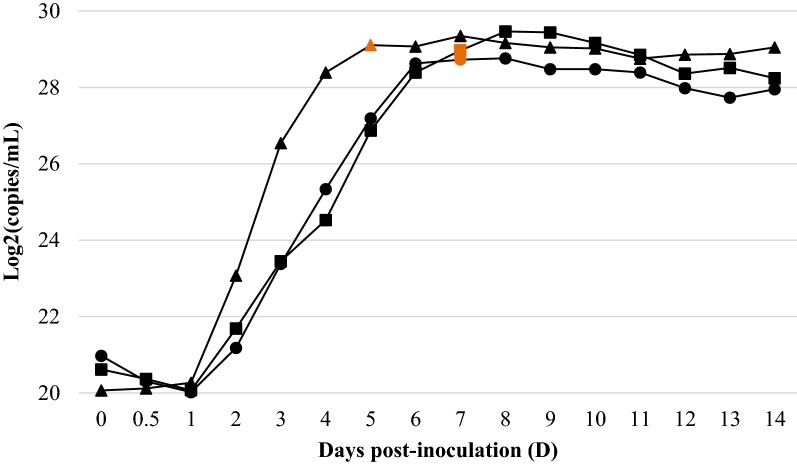
***Mycoplasma hyopneumoniae*** **strains J (filled triangle), 11 (filled square) and 232 (filled circle) copies per millilitre.** Data points represent means of the 2 replicate qPCR measurements (A and B) within each strain. The orange data points indicate when the original medium colour change was first visually detected in each strain culture.

*Mycoplasma hyopneumoniae* genome copy numbers per mL of the three strain cultures assessed by the fluorescent dsDNA stain assay and qPCR are shown in Figures 5 and 6, respectively. Both methodologies resulted in similar curves from which a lag and log phases could be recognised. However, the range of copy numbers assessed by qPCR was wider than the one assessed by fluorimetry. Once reached the maximal number of *M. hyopneumoniae* copies/mL, a slightly gradual decrease in the copy numbers was observed as time progressed, though stationary and senescence phases could not be differentiated. Remarkably, the reach of the maximal number of *M. hyopneumoniae* copies/mL of culture concurred approximately in time with the maximal ATP values.

### Growth kinetic parameters of *M. hyopneumoniae*

The Gompertz model could not be reliably applied to CFU titres because of the lack of data for 11 and 232 *M. hyopneumoniae* strains and the very short log phase as well as the lack of a stationary phase of the J strain growth curve. For all other techniques, the values of the parameters estimated for the Gompertz model are shown in Table 1. All three strains had close values for parameters *A* and *B* in each of the evaluated techniques but for qPCR, in which significant differences between 11 and 232 strains were observed. Remarkably, the kinetic parameters *C*, *μ*_max_ and G obtained from J culture were significantly lower (*p* < 0.05) than the ones corresponding to the 11 strain culture for all the assessed techniques but for CCU. Indeed, none of the parameters obtained from the application of the Gompertz model to the CCU data was significantly different between *M. hyopneumoniae* strains.

**Table 1:** **Parameter estimates, coefficient of determination (** ***R*** ^**2**^ **) and average prediction error (APE) for Gompertz model used to describe** ***M. hyopneumoniae*** **strains J, 11 and 232 growths**

Technique	Strain	*A* ^a^	*B* ^a^	*C* ^b^	*μ*_*max*_^c^	*G* ^d^	R^2^	APE^e^
ATP luminometry	J	1493.868	10.626	1.065ª	1.171ª	14.607ª	0.981	−5.118
	11	1677.066	15.485	1.042^b^	1.121^b^	20.317^b^	0.983	5.423
	232	1497.592	15.909	1.056^ab^	1.159^ab^	15.756^ab^	0.985	−3.054
CCU	J	1.25E+10	1.47E+02	1.083	1.310	9.536	0.938	−34.413
	11	9.58E+09	1.48E+03	1.055	1.205	13.788	0.897	−5.401
	232	1.51E+10	5.79E+00	1.026	1.175	14.649	0.829	−70.113
Fluorimetry	J	2.18E+08	1.54E+04	1.143ª	1.228ª	11.610ª	0.997	−4.773
	11	2.54E+08	1.92E+03	1.079^b^	1.128^b^	19.256^b^	0.991	−2.233
	232	1.69E+08	3.23E+04	1.115^ab^	1.160^ab^	15.585^ab^	0.991	−5.161
qPCR	J	7.46E+08^ab^	2.67E+02^ab^	1.110ª	1.331ª	8.162ª	0.997	0.074
	11	8.01E+08ª	2.27E+02ª	1.068^b^	1.185^b^	13.597^b^	0.986	−6.814
	232	4.98E+08^b^	2.17E+04^b^	1.103^ab^	1.252^ab^	10.535^ab^	0.983	−12.984

Different superscripts within a column and technique indicate significant differences between strains (*p* < 0.05).

^a^Pmol ATP, CCU or copies of *M. hyopneumoniae* per mL.

^b^Pmol ATP, CCU or copies of *M. hyopneumoniae* per mL per hour per unit of maximal titre (parameter *A*).

^c^Pmol ATP, CCU or copies of *M. hyopneumoniae* per mL per hour.

^d^Time (hours).

^e^Percentage (%).

**Figure 7:**
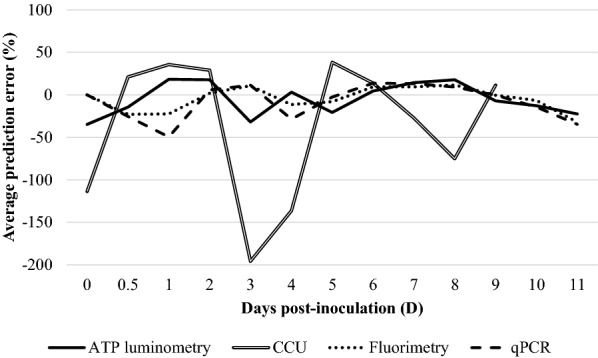
**Distribution of average prediction error (APE) of** ***M. hyopneumoniae*** **titre data from distinct titration techniques.** APE was defined by the Gompertz model for data obtained from the application of ATP luminometry, CCU, fluorimetry and qPCR techniques.

All Gompertz regression models had *p* values lower than 0.05 and *R*^2^ values between 0.83 and 1. In general terms, mean APE values of each strain and technique were negative, indicating overall overestimated predictions by the model. Calculated mean APE technique values (average from all strain APE within a method) are shown in Figure 7. APE associated with prediction of *M. hyopneumoniae* culture titre at each time showed that differences between predicted and actual titre values alternated in positive and negative sign in all procedures. The highest error, however, was observed in the CCU method. For all the other assessed techniques.

### Relationship between ATP, CCU, fluorimetry and qPCR assays

Similar to what happened for the Gompertz modelling, the lack of CFU data hampered the comparison of this technique with the others. During the exponential growth phase, there was high correlation (r > 0.9) between all the other evaluated methodologies in all three *M. hyopneumoniae* strain cultures and these were statistically associated (*p* < 0.001) (Table 2). Equivalences between the assessed methods resulting from the application of the formulae from the linear regression analyses between each pair of techniques in each strain culture are provided in Table 3.

**Table 2:** **Correlation between evaluated techniques considering data from the logarithmic phase of growth (according to ATP luminometry assay) of** ***M. hyopneumoniae*** **strain J, 11 and 232 cultures**

Strain	Technique			
	ATP	CCU	Fluorimetry	qPCR
ATP				
J	–	0.929	0.930	0.961
11	–	0.924	0.952	0.966
232	–	0.950	0.945	0.966
CCU				
J	–	–	0.958	0.954
11	–	–	0.985	0.979
232	–	–	0.933	0.925
Fluorimetry				
J	–	–	–	0.982
11	–	–	–	0.993
232	–	–	–	0.989

All systems were statistically associated (*p* < 0.001).

**Table 3:** **Values of each evaluated technique corresponding to a unit of a particular method (first column) in each** ***M. hyopneumoniae*** **strain culture**

Technique (one unit)	Strain	Technique (equivalence units)			
		ATP (pmol ATP/mL)	CCU (CCU/mL)	Fluorimetry (*M. hyopneumoniae* copies/mL)	qPCR (*M. hyopneumoniae* copies/mL)
ATP (pmol ATP/mL)	J	–	1.76E+05	6.09E+05	6.40E+04
	11	–	4.05E+05	1.23E+06	2.09E+05
	232	–	1.66E+03	1.08E+06	1.71E+05
	Mean (± SD)	–	1.94E+05 (± 2.02E+05)	9.72E+05 (± 3.23E+05)	1.48E+05 (± 7.51E+04)
CCU (CCU/mL)	J	2.10E−04	–	9.68E+02	3.79E+00
	11	3.92E−05	–	1.02E+03	4.65E+00
	232	1.23E−02	–	7.79E+04	3.77E+03
	Mean (± SD)	4.17E−03 (± 7.00E−03)	–	2.66E+04 (± 4.44E+04)	1.26E+03 (± 2.17E+03)
Fluorimetry (*M. hyopneumoniae* copies/mL)	J	7.99E−08	< 1	–	< 1
	11	6.47E−09	< 1	–	< 1
	232	1.53E−09	< 1	–	< 1
	Mean (± SD)	2.93E−08 (± 4.39E−08)	NA	–	NA
qPCR (*M. hyopneumoniae* copies/mL)	J	2.12E−04	< 1	3.50E+02	–
	11	2.51E−05	< 1	3.69E+02	–
	232	1.18E−05	< 1	3.20E+02	–
	Mean (± SD)	8.30E−05 (± 1.12E−04)	NA	3.47E+02 (± 2.49E+01)	–

Equivalences were obtained by fitting the linear regression models ofeach pair of techniques comparison to data from the maximal culture growth (log phase).

## Discussion

In the present study, the course of the in vitro growth of *M. hyopneumoniae* J, 11 and 232 strains was evaluated by the application of different techniques with the potential of being used as an alternative to CCU for *M. hyopneumoniae* culture titration. Growth curve kinetics and dynamics of *M. hyopneumoniae* J strain were different from the observed for 11 and 232 strains, which, in turn, showed very similar growth curves regardless the technique applied. Thus, growth curve plots revealed a faster in vitro growth of the J strain in comparison with 11 and 232 strains as well as a straighter log phase, and a shorter stationary phase followed by a steeper senescence phase. Nonetheless, raw data analysis showed no differences between strains in terms of maximal titres reached within a particular technique.

Results obtained by those methods that indirectly measure the number of metabolising cells (i.e. CCU and ATP assays) indicated that, once maximal levels were reached, CCU titres dropped earlier than the onset of decrease in ATP titres. This fact supports that *M. hyopneumoniae* loses its capacity to divide (requisite for the CCU titration) before cell lysis and intracellular ATP impairment [13, 27]. Considering ATP growth curves, the medium colour change from red to orange was observed in the last section of the log phase, whereas the shift to yellow was complete in the stationary phase. However, when CCU growth curves are taken into account, the original red shift to orange happened when all strain cultures were already in the stationary phase. Henceforth, *M. hyopneumoniae* growth phase in which medium turn colour may vary depending on different conditions, such as the medium itself or even the batch of medium used [13], the volume of medium to be judged, as well as on the technique used to assess the growth of the bacterium. Therefore, extrapolation of the growth phase from the colour of the medium should be used with caution. On the other hand, both molecular methods (i.e. fluorimetry and qPCR assays) led to very similar growth curve responses, showing a slightly gradual decrease in the copy numbers once reached the stationary phase. This finding indicates that DNA fragments might be present in culture for longer periods once cells are dead. Henceforth, extrapolation of the growth phase from genomic copy numbers can also be confounding as no clear differentiation could be established between stationary and senesce phases.

In the present work, *M. hyopneumoniae* colonies were seen for all three strains assessed. Nevertheless, a growth curve based on CFU could only be determined by J strain, since growth of 11 and 232 colonies was much more limited. These results indicate that solid medium might support differently the growth of diverse *M. hyopneumoniae* strains. Indeed, *M. hyopneumoniae* growth on solid medium has been considered particularly difficult [28] and solidification of Friis medium with agar may hamper the growth of colonies of *M. hyopneumoniae*, suggesting that agar inhibits the growth or sequesters essential nutrients for growing [29]. In a recently published work, however, *M. hyopneumoniae* growth curve could be accurately determined by using viable counts on the solid medium [29] and such curve was, actually, similar in shape to the one obtained for strain J in the present study. Strain J culture reached a maximum viability of 6.5 × 10^5^ CFU/mL at D6, which approximately corresponded to the medium colour shift to orange as well as with the end of the log phase determined by ATP culture concentrations. After this point, there was a decline until D10, from where no more colonies were observed. For 11 and 232 strains, colonies appeared when cultures were in the stationary phase (data not shown). Although depending probably on several conditions such as the *M. hyopneumoniae* strain or the medium used, overall, obtained results suggest that the CFU technique underestimates the number of live cells, suggesting the inappropriateness of this method for *M. hyopneumoniae* titration.

Microbial growth curve is typically depicted in terms of log numbers and it has a characteristic sigmoid shape. Thus, one way to describe bacteria growth is using nonlinear regression models, such as the Gompertz equation [23, 30]. This model accommodates measurements in some kinetics parameters and permits appropriate biological interpretation. In the present case and based on the *R*^2^, the Gompertz model was well adjusted to data coming from the application of different titration techniques in all three strain cultures, except for the CFU. As a reference, former application of the Gompertz model to *M. hyopneumoniae* ATP growth curves resulted also in high *R*^2^ values [13]. Overall, from the comparison between APE values obtained for each technique, the Gompertz model had poorer fit during the early than at late phases of *M. hyopneumoniae* growth. In the present study, the lack of data at early phases (hours subsequent to the initial inoculum dilution) was perhaps a major limitation of this model. Moreover, CCU was the worst predicted method by the model, probably because progression along time did not always correspond to a further dilution turning colour, not showing a typical bacteria growth curve.

Information related to the association between the in vitro growth and the difference in pathogenicity or virulence between *M. hyopneumoniae* strains is scarce and can often be difficult to interpret. In fact, the information available on such respect is up to now contradictory [10–13]. In the present study, estimated kinetic parameters by the Gompertz model confirmed the faster in vitro performance of the non-pathogenic J strain observed in the growth curve plots. Indeed, all the applied methods mostly agreed that J was the fastest growing strain and the pathogenic 11 the slowest one. In agreement, *M. hyopneumoniae* J strain protein expression profile was found to be related to metabolism (indicative of a non-infective proliferate lifestyle that would fit a better adaptation to the in vitro conditions) whereas proteins expressed by pathogenic strains (e.g. 232 strain) were predicted to be more associated to the infectious competence [10, 11]. This would also agree with the described loss of virulence and adhesion capacity due to successive in vitro passages [3, 14, 15]. In contradiction to obtained results, a faster in vitro multiplication (determined by the CCU assay) was observed in a highly virulent *M. hyopneumoniae* isolate when compared to a low virulent one [12]. Later on, however, the same research group studied the course of growth of those two isolates, together with other isolates proved to be diverse in virulence [31], genetically [32] and phenotypically [33] and did not find association between isolates virulence and growth response curves assessed by ATP luminometry [13]. Overall results suggest a putative relationship between virulence and the in vitro growing capacity of *M. hyopneumoniae*, although further studies are needed to confirm results and reinforce this hypothesis.

Although reasons for the former inconsistencies are difficult to elucidate, some aspects should be kept in mind. First, the growth medium has been reported to have a more pronounced effect on the *M. hyopneumoniae* growth response than the isolate by itself [13]. Second, comparisons of the course of growth of different *M. hyopneumoniae* are hindered by the inherent difficulty in obtaining strain initial inoculum with the same amount of *M. hyopneumoniae* live cells. In the present case, all strain initial inoculum came from single colonies and were used when reached similar maximal ATP values. Moreover, such inocula were equally diluted at D0 and volumes taken at every time point were the same for all strain cultures. The abovementioned aspects may also partly explain the differences found in the kinetics of the growth curves between this work and a previous one [13]. While the initial ATP and CCU concentrations were fairly similar between both studies, the former reported growth curves were much shorter in time than the ones reported in here. Although other many growing conditions might be implicated in such differences, this finding highlights again the effect that the use of different culture media may have on *M. hyopneumoniae* in vitro growth. Besides dissimilarities, it is important to note that previous estimated maximum ATP titres reached values of 1350 pmol ATP/mL [13], which were not distant from the maximum values appraised in this work of 1689 pmol ATP/mL.

Comparison of results obtained by the different applied techniques was an important part of the present study. During the log growth phase, titration results of the four assays were highly and significantly correlated and a strong linear relationship was observed. Ideally, further *M. hyopneumoniae* strains as well as culturing time points should be included to better assess the relationship between techniques. Nevertheless, growth kinetics of 7 diverse *M. hyopneumoniae* isolates were previously judged and, in agreement with the present results, CCU and ATP titres were strongly linearly linked during log phase [13]. According to the assessed regression models, *M. hyopneumoniae* culture had a mean ATP content of 4.17 × 10^−3^ pmol per colour change of 1 mL culture, whereas another study reported *M. hyopneumoniae* culture to contain 1.77 × 10^−6^ pmol ATP per CCU in 1 mL of culture [13]. Henceforth, in the present work, a higher amount of ATP was needed in comparison to the previous one to achieve the change of colour of 1 mL culture, which may partly explain the different lengths between growth curves of both studies. Neither CCU, ATP nor fluorimetry assays are specific for *M. hyopneumoniae*, which might be problematic when other inoculum type rather than culture is used (e.g. lung homogenate). In this case, *M. hyopneumoniae* copies per mL assessed by qPCR may be the most approximated value to the number of *M. hyopneumoniae* cells. The amount of *M. hyopneumoniae* DNA determined by qPCR revealed that 1 CCU/mL correspond to 1260 mycoplasmas. This value is close to previous data reporting that 1 CCU/mL of *M. hyopneumoniae* strain 116 corresponded to 1000 mycoplasmas [20]. While qPCR might be a reliable titration technique during the *M. hyopneumoniae* log growing phase, its inability to differentiate between alive and dead cells is a major disadvantage during the later stages of the growth. Given that each of the evaluated titration methodologies have limitations, their use in combination is probably required to optimise *M. hyopneumoniae* culture titration accuracy.

In order to standardize *M. hyopneumoniae* growth protocols in the laboratory as well as inoculum production for experimental applications, it is important to know the in vitro growth kinetics and dynamics of a particular strain in a particular medium. Once this information is known, a more reliable approximation of the growing phase status from the culture colour change can be performed. For this purpose, ATP is an economical, rapid, timely and efficient assay that allows the distinction of the different *M. hyopneumoniae* growing phases in culture. Nonetheless, ATP is not a *M. hyopneumoniae* specific technique; thus, its combination with a specific detection method (such the qPCR) might be useful. Lastly, further studies with more strains, both at in vitro and in vivo levels, are needed to elucidate the putative relationship between *M. hyopneumoniae* in vitro behaviour and virulence.

## Abbreviations

*M. hyopneumoniae*: *Mycoplasma hyopneumoniae*
CCU: colour changing units
CFU: colony forming units
PCR: polymerase chain reaction
qPCR: real time quantitative PCR
dsDNA: double-stranded DNA
G: generation time
ATCC: American Type Culture Collection
D: days post-inoculation
RLU: relative light units
RFU: relative fluorescence units
*μ*_max_: maximum growth rate
APE: average prediction error
*Y*: observed titre values
*PY*: predicted titre values
